# Melt Amorphisation of Orlistat with Mesoporous Silica Using a Supercritical Carbon Dioxide: Effects of Pressure, Temperature, and Drug Loading Ratio and Comparison with Other Conventional Amorphisation Methods

**DOI:** 10.3390/pharmaceutics12040377

**Published:** 2020-04-20

**Authors:** Heejun Park, Kwang-Ho Cha, Seung Hyeon Hong, Sharif Md Abuzar, Eun-Sol Ha, Jeong-Soo Kim, Min-Soo Kim, Sung-Joo Hwang

**Affiliations:** 1 College of Pharmacy, Pusan National University, 63 Busandaehak-ro, Geumjeong-gu, Busan 46241, Korea; pharmacy4336@pusan.ac.kr (H.P.); edel@pusan.ac.kr (E.-S.H.); 2 Yonsei Institute of Pharmaceutical Sciences & College of Pharmacy, Yonsei University, 85 Songdogwahak-ro, Yeonsu-gu, Incheon 21983, Korea; horizon0712@gmail.com (K.-H.C.); shhongmartin@naver.com (S.H.H.); sumonzar@gmail.com (S.M.A.); 3 Dong-A ST Co. Ltd., Giheung-gu, Yongin, Gyeonggi 446-905, Korea; ttung2nd@naver.com

**Keywords:** orlistat, mesoporous silica, supercritical carbon dioxide, melt-amorphisation, dissolution

## Abstract

The aim of this work was to develop an amorphous orlistat-loaded mesoporus silica formulation using the melt-amorphisation by supercritical fluid (MA-SCF) and to investigate the effects of pressure and temperature on the pharmaceutical properties of the developed formulation. In addition, the effect of orlistat mass ratio to the mesoporus silica was also evaluated. The carbon dioxide was used as a supercritical fluid, and Neusilin^®^UFL2 was selected as the mesoporous silica. For comparison with conventional amorphisation methods, orlistat formulations were also prepared by solvent evaporation and hot melt methods. Various pharmaceutical evaluations including differential scanning calorimetry, powder X-ray diffraction, scanning electron microscopy, specific surface area, total pore volume, and content uniformity were performed to characterise the prepared orlistat formulation. The melting point depression and the solubility of orlistat in supercritical carbon dioxide (SC-CO_2_) were selected for the interpretation of evaluated results in relation to temperature and pressure. The total pore volume of the prepared orlistat-loaded mesoporus silica decreased with an increasing density of SC-CO_2_ to about 500 g/L at a constant temperature or pressure. From these results, it was suggested that increasing the density of SC-CO_2_ to about 500 g/L could result in the easier penetration of CO_2_ into molten orlistat and lower viscosity, hence facilitating the introduction and loading of orlistat into the pores of Neusilin^®^UFL2. However, when the density of SC-CO_2_ increased to more than 500 g/L, the total pore volume increased, and this may be due to the release out of orlistat from the pores of Neusilin^®^UFL2 by the increased orlistat solubility in SC-CO_2_. Interestingly, as the total pore volume decreased by the filling of the drug, the drug crystallinity decreased; hence, the dissolution rate increased. Furthermore, it was shown that the most desirable mass ratio of Neusilin^®^UFL2:orlistat for the amorphisation was 1:0.8 at an optimised supercritical condition of 318 K and 10 MPa. Compared with other amorphisation methods, only the sample prepared by the MA-SCF method was in pure amorphous state with the fastest dissolution rate. Therefore, it was concluded that the amorphous orlistat-loaded mesoporus silica prepared using MA-SCF under optimised conditions was more advantageous for enhancing the dissolution rate of orlistat than other conventional amorphisation methods.

## 1. Introduction

**Figure 1:**
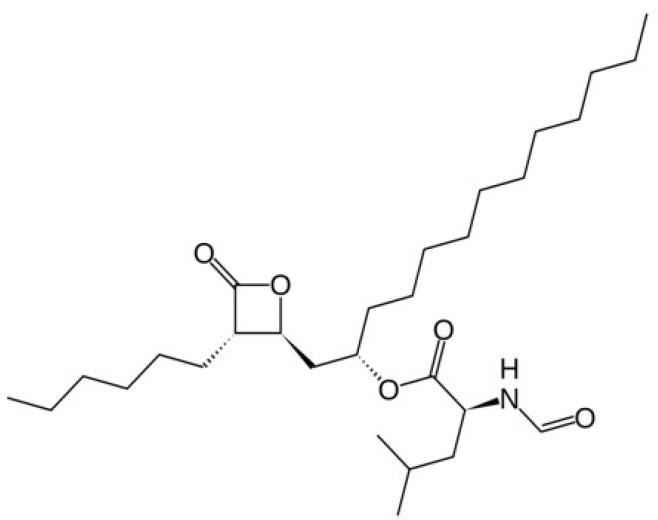
Chemical structure of orlistat.

Orlistat, a derivative of lipstatin, is a potent lipase inhibitor isolated from the bacterium *Streptomyces toxytricini* and has been used for many years to treat obesity. The chemical name of orlistat is (S)-1-[[(2S,3S)-3-hexyl-4-oxo-2-oxetanyl]methyl]-dodecyl ester (Figure 1). It has been approved for use in many countries, and its pharmacological activity is well understood [1,2,3]. Orlistat works by inhibiting the gastric and pancreatic lipases, which are the enzymes that break down triglycerides in the intestine. When lipase activity is blocked, triglycerides from food are not hydrolysed into absorbable free fatty acids but are excreted undigested [4]. Only trace amounts of orlistat are absorbed systemically, and the primary route of elimination is through the feces. Due to this, undigested lipids in the bowel lumen stimulate contractility, which in turn results in gastrointestinal side effects such as fecal incontinence, oily spotting, flatus, fecal urgency, oily stools, and increased defecation [5].

One major challenge associated with using orlistat is its low solubility. According to the biopharmaceutics classification system, orlistat is a class II low-solubility and high-permeability drug [6]. The slow dissolution rate of orlistat limits its pharmacological activity. Several studies have been carried out to enhance the dissolution rate of orlistat using various drug delivery systems, such as nanoparticles [7,8], solid lipid nanoparticles [9], solid dispersions [10,11,12], self-emulsifying drug delivery systems [13,14,15], liquisolid formulations [16], and dry-foam tablets [17]. However, the use of these drug delivery platforms often presents problems such as formulation instability, complexity of the manufacturing process, and difficulty in commercialisation [18]. Particularly, the handling of micronized or nanosized drugs is often problematic because small particles tend to agglomerate with high surface energy; hence, an additional stabilisation process is usually required during the commercial manufacturing process. A method of improving the drug dissolution rate, while avoiding this difficulty, is to adsorb the drug onto a mesoporous carrier [19,20,21]. In this technique, the drug is dissolved in an organic solvent and a mesoporous silica carrier is added to this solution. The agglomeration of drug particles is prevented by the binding of drug to the carrier during solvent removal. However, the residual organic solvent in the drug formulation, which is present even after the completion of the solvent removal process, may cause some problems [22,23]. To address this issue, several studies have reported the use of supercritical fluid (SCF) technology to replace the organic solvent.

An SCF is defined as a substance with a single fluid phase above its critical temperature and pressure, wherein it can dissolve materials similar to a liquid and diffuse through solids as a gas [24,25,26]. The advantageous properties of SCF, such as solvent power, viscosity, and diffusivity can be easily controlled by manipulating the temperature and pressure. SCFs have been used in the pharmaceutical industry as a suitable substitute for organic solvents [27,28,29]. Supercritical carbon dioxide (SC-CO_2_) is the most widely used SCF, owing to its mild critical conditions, non-toxicity, non-flammability, and low cost. Many researchers have employed SCF technology to modify the solid-state properties of active pharmaceutical ingredients, such as polymorphism and crystallinity, which affect their dissolution rate and bioavailability [30]. Sanganwar et al. reported that SC-CO_2_ can be successfully used as a solvent for the adsorption of fenofibrate onto silica to enhance its dissolution [31]. On the other hand, Cha et al. used supercritical CO_2_ to lower both the melting temperature of fenofibrate and the viscosity of molten fenofibrate, and thereby load the molten fenofibrate into the adsorbent as an amorphous composite [32]. These studies took advantage of the fact that gases with high solubility can considerably decrease the melting points of chemical drugs [33,34,35]. However, the former supercritical adsorption method requires the drug to be dissolved in SC-CO_2_, but the latter supercritical melting method requires melting of the drug rather than dissolving in SC-CO_2_. SC-CO_2_ can diffuse well into a solute, lowering its melting point, viscosity, and surface tension; this in turn aids the penetration of the substrate into the pores of mesoporous silica, and the melted drug can then be easily distributed into the pores of mesoporous silica [36,37,38]. Thus, this melt-amorphisation by supercritical fluid (MA-SCF) has less restriction due to the limited solubility of the drug in SC-CO_2_, and it is possible to prepare a formulation with high drug loading.

In this study, the MA-SCF was used to prepare an amorphous orlistat-loaded mesoporous silica and the effects of pressure and temperature on the loading behavior of the drug on mesoporous silica were evaluated. Neusilin^®^UFL2, a synthetic amorphous form of magnesium aluminometasilicate with high surface area, large mesopore volume, narrow mesopore size distribution (5-8 nm), and ordered unidirectional mesopore network was selected as the solid carrier of mesoporous silica type. The carbon dioxide was used as a supercritical fluid. To the best of our knowledge, there have been no attempts to apply the MA-SCF for enhancement of the dissolution rate of orlistat. The melting point depression of orlistat in SC-CO_2_ was determined to establish operating conditions for the MA-SCF process. The density of SC-CO_2_ and the solubility of orlistat in SC-CO_2_ were investigated to better understand the changes in loading behavior due to process variables such as the temperature and pressure. In addition, the effect of the drug mass ratio was also evaluated. For comparison with conventional amorphisation methods, orlistat formulations were also prepared by solvent evaporation (SE) and hot melt (HM) methods. The prepared orlistat formulations were characterised by differential scanning calorimetry (DSC), powder X-ray diffraction (PXRD), scanning electron microscopy (SEM), Brunauer–Emmett–Teller (BET) specific surface area analysis, total pore volume analysis, and content uniformity analysis. The powder dissolution test was also conducted to evaluate the differences in dissolution rates between samples.

## 2. Materials and Methods

### 2.1. Materials

Orlistat was obtained from Biocon Co., Ltd. Neusilin^®^UFL2 was purchased from Fuji Chemical Industry Co., Ltd. (Toyama, Japan). Phosphoric acid and sodium lauryl sulfate (SLS) were supplied from Samchun Pure Chemical Co., Ltd. (Pyeongtaek, Korea) and Ducsan Co., Ltd. (Ansan, Korea), respectively. CO_2_ (99.99% purity) was purchased from Hanmi Gas Co., Ltd. (Daejeon, Korea). All other solvents were of high-performance liquid chromatography (HPLC) grade (Fisher Scientific, Pittsburgh, PA, USA). All other chemicals were of reagent grade.

### 2.2. Measurement of Orlistat Solubility in SC-CO_2_

**Figure 2:**
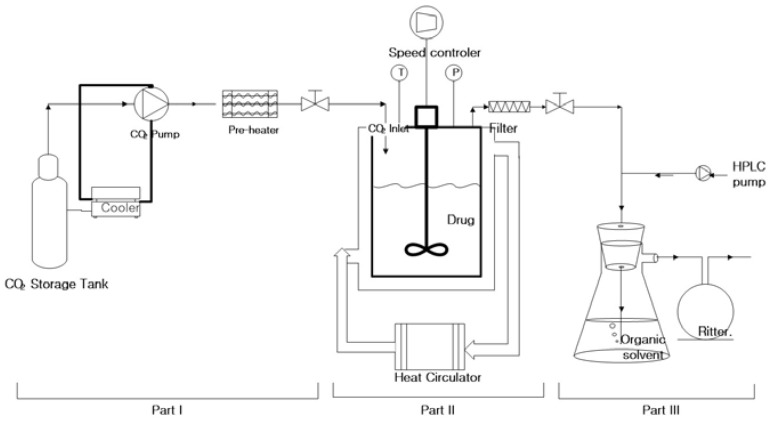
Schematic diagram of the apparatus for measurement of solubility in SC-CO_2_.

The solubility of orlistat was measured over a pressure range from 8 to 16 MPa at 308.15, 313.15, and 318.15 K (Table 1), using a high-pressure apparatus equipped with a sapphire window, which was made according to the procedure described by Su et al. [39]. As shown in Figure 2, the experimental system included three parts: (I) the SC-CO_2_ feeding part, (II) the reaction cell part to maintain equilibrium between the solid drug and the supercritical phases, and (III) the expansion part to capture the drug in the organic solvent for the quantitative analysis of the drug. To check the reliability of the apparatus, the determination of solubility of fenofibrate was carried out at 318.2 K in the 10–18 MPa pressure range, since the solubility of this compound in the supercritical CO_2_ is well studied. As shown in Figure S1 (Supplementary Material), the solubility values of fenofibrate obtained in this work are in good agreement with reported data by Chen et al. [40]. These results confirmed that the apparatus employed in this work for the measurement of drug solubility in SC-CO_2_ is reliable. The solubility of orlistat was determined by the following procedure. The reaction cell in part 2 was sealed after loading an excess amount of orlistat. The enclosed air was removed from the cell by allowing small amounts of CO_2_ in and out of the cell several times. Compressed CO_2_ was delivered to the cell through a pre-heater by the ISCO syringe pump (Model 260D, Lincoln, NE, USA) that was included in part 1. The temperature was maintained by a water-circulating jacket around the cell (part 2). After reaching the equilibrium cell, SC-CO_2_ was expanded into atmospheric pressure through a 0.5 µm line filter (HIP, Erie, PA, USA) and needle valve, where they were located at the end of part 2. The needle valve was wrapped by the heating tape to keep in the temperature range of about 10 K above the melting point of the drug for the prevention of precipitation and blockage in the line. At the same time, the pressure was regulated constantly by the ISCO pump with a constant pressure function and a back pressure regulator. Then, the drug was separated from the gas phase and dissolved into an organic solvent in a flask (part 3). The residual drug in the line was recovered by additional washing using an organic solvent. The total volume of CO_2_ flow was measured by a wet test meter (Ritter TG05, Bochum, Germany) and confirmed by comparison with the volume of injected CO_2_ from the compression cylinder at a constant temperature displayed on the ISCO pump. The total amount of CO_2_ was calculated using the density of CO_2_. The density values for CO_2_ were obtained from the NIST REFPROP database [41]. The amount of orlistat that dissolved in a measured volume of SC-CO_2_ was determined by HPLC analysis of the orlistat trapped in the organic solution. The solubility experiment at each condition was triplicated.

### 2.3. Correlation of Density of SC-CO_2_ and Orlistat Solubility in SC-CO_2_ by Semi-Empirical Models

Two semi-empirical equations, presented by Chrastil and Mendez-Santiago and Teja (MST), were employed to correlate the density of SC-CO_2_ and orlistat solubility in SC-CO_2_ [42,43]. The Chrastil model is expressed by a linear relationship between the logarithm of the solid solubility and the logarithm of the density of pure CO_2_, using the following equation:

ln*y* = *k*ln*ρ* + *a*/*T* + *b*, (1)

where *y* is the solubility of orlistat in SCF (g/L), *ρ* is the density of SC-CO_2_ (g/L), and *T* is the temperature (K). The association number *k* and the other two constants *a* and *b* were taken as three empirically fitted model parameters.

Another semi-empirical equation including the MST model is based on the theory of dilute solutions. The MST equation can be expressed as follows:

*T*ln(*yP*) = *c* + *dρ* + *eT*, (2)

where *c*, *d,* and *e* are regression parameters, *y* is the solubility of orlistat in SCF (mole fraction), *ρ* is the density of SC-CO_2_ (g/L), *P* is the system pressure (MPa), and *T* is the system temperature (K). The parameters mentioned above were calculated by correlating the experimentally obtained results. In order to obtain the parameters, multiple linear regression analysis was performed based on Equations (1) and (2), using the SPSS 12.0 software (IBM SPSS, Chicago, IL, USA). The average absolute relative deviations (AARD) were calculated by the following Equation (3):

(3) $$\mathrm{AARD}(\%)=(100/n)\;\sum_{k=1}^{n}\frac{y_{k}^{\exp}-y_{k}^{\mathrm{cal}}}{y_{k}^{\exp}},$$

where *n* is the number of experimental points and *y*_k_^exp^ and *y*_k_^cal^ (mole fractions) are the solubilities of orlistat in SCF, obtained experimentally and by calculation using a prediction model, respectively.

### 2.4. Determination of Melting Point Depression by Solid–Liquid (S-L) Phase Behavior Experiment

A high-pressure variable-volume view cell, equipped with a sapphire window, pressure transducer and thermocouple, was used to determine the melting point of orlistat in a binary system with CO_2_, as described in our previous study [32]. Each experiment was triplicated. The temperature was controlled by the combination of a water-circulating jacket around the cell and by placing the equipment in an air convection oven. The experiments were carried out over a temperature range of 288.15 to 313.15 K, as presented in Table 2. An excess amount of raw orlistat was placed inside a glass tube (i.d.= 1.2 mm), and the glass tube was put into the cell and the cell was sealed. The compressed CO_2_ was delivered through a pre-heater into the cell by an ISCO pump. After the removal of air from the cell, CO_2_ was added at a constant temperature, until the desired relatively low initial pressure was reached. After stabilisation of the initial pressure at a constant temperature, the pressure was adjusted by the change in volume inside the cell by moving the piston. As the pressure was increased at a fixed temperature, the phase behavior of orlistat in the glass tube was monitored through a window by a video camera. The melting point of orlistat was determined by visual observation when the first droplet of the molten liquid was formed. After completing the measurement of melting point at a temperature, the entire process was repeated at another temperature. The melting temperature depression (ΔT_m_) at pressurised condition (T_m_p_) compared to the onset temperature of melting endothermic peak in the DSC thermogram of raw orlistat at ambient pressure (T_m_a_), was calculated by the following Equation (4):

ΔT_m_ = T_m_p_ − T_m_a_. (4)

### 2.5. Drug Quantification Method

The HPLC-ultraviolet (UV) method was used to determine the orlistat concentration in the formulation. The HPLC system consisted of a WatersTM 2690 Alliance analytical HPLC, including an auto-sampler and a Waters^TM^ 996 photodiode-array UV detector (Waters, Milford, MA, USA). The analytical column was Xterra^TM^ RP-18 (150 × 4.6 mm, 5 µm, Waters, Milford, MA, USA). The mobile phase consisted of acetonitrile:water (95:5, *v/v*) with 0.1% (*v/v*) phosphoric acid, and the flow rate was 1 mL/min. The UV-detection wavelength was set at 205 nm. The data acquisition and evaluation were performed with Millennium 32 software (Waters, Milford, MA, USA).

### 2.6. Preparation of Amorphous Orlistat-Loaded Neusilin^®^ UFL2 Using MA-SCF Process

Neusilin^®^UFL2 was selected as the solid carrier of mesoporous silica type because of its large pore volume (1.41 ± 0.06 cm^3^/g) and specific surface area (403.93 m^2^/g). The equipment for MA-SCF was similar to the one described in our earlier study, except that a vessel of 60 mL volume was used. Orlistat was weighed accurately and fully mixed with Neusilin^®^UFL2 in a tumble mixer, and then the initial mixture was placed at the bottom of a stainless steel high-pressure vessel. Subsequently, the vessel was sealed and compressed CO_2_ was delivered inside the vessel using the ISCO syringe pump (Model 260D, Lincoln, NE, USA), until the desired pressure was reached. The temperature of the vessel was controlled by a program type of a refrigerated and heating bath circulator (RW3-3035P, JeioTech, Daejeon, Korea). The system was maintained at a constant pressure and temperature for 90 min to load the melted orlistat onto the pore of the Neusilin^®^UFL2. Next, the vessel was depressurised at an approximate rate of 0.35 ± 0.05 MPa/min. Simultaneously, the temperature of vessel was cooled to 298 K at a programmed constant cooling rate using a bath circulator until the atmospheric pressure is reached. Finally, the prepared powder samples were collected from the vessel.

#### 2.6.1. Effect of Pressure and Temperature

The mass ratio of Neusilin^®^UFL2:orlistat = 1:1.2 was chosen, taking into consideration the estimated maximum theoretical loading capacity of orlistat inside the pore channel, based on the lowest value of pore volume (1.41 ± 0.06 cm^3^/g) measured three times and true density of amorphous orlistat (0.92 g/cm^3^, for crystalline 0.98 g/cm^3^). The experimental conditions are shown in Table 3. The preliminary study confirmed that the samples were obtained under sub-critical conditions where CO_2_ can exist in two different phase states, a liquid or a gaseous state, as well as in two-phase mixtures of these states at lower temperatures and/or pressures than 304.1 K and 7.4 MPa of supercritical condition; these states were very highly crystalline, and thus it was difficult to produce amorphous orlistat-loaded solid composite under these conditions. Regarding the preliminary result, this study designed experiments within the temperature and pressure range of supercritical conditions. In addition, based on the results from the melting point depression test, it was confirmed that orlistat was molten within the conditions used.

#### 2.6.2. Effect of Mesoporous Silica:Drug Mass Ratio

Orlistat-loaded Neusilin^®^UFL2 with different mass ratios were prepared at a fixed process condition to obtain the optimum loading ratio (Table 3). The mass ratios of Neusilin^®^UFL2:orlistat used were 1:1.2, 1:1, and 1:0.8 (*w/w*).

### 2.7. SE Method

Orlistat (400 mg) was dissolved in 60 mL of ethanol, and 500 mg of Neusilin^®^UFL2 was then added to the orlistat solution. The suspension was stirred for 12 h to achieve equilibrium, and then ethanol was evaporated using a rotary evaporator at 40 °C.

### 2.8. HM Method

Four hundred milligrams of orlistat were fully mixed with 500 mg of Neusilin^®^UFL2 using a tumble mixer. The mixture was heated at 80 °C using MSH-30D digital heating-plate (Wisestir^®^, Daihan scientific Co., Ltd., Wonju, Korea) to melt the orlistat, which was then cooled to 25 °C in a drying desiccator for solidification.

### 2.9. Specific Surface Area and Total Pore Volume

The specific surface area of the prepared powder was determined using a surface area and pore size analyser (ASAP 2010, Micromeritics, Norcross, GA, USA). The sample powders were placed in tubes and weighed; then, they were degassed at 25 °C (below melting point of orlistat) for at least 24 h under nitrogen gas purge using a FlowPrep 060 degasser (Micromeritics, Norcross, GA, USA). The experiments were started after confirmation that there is no more change in the mass of the sample. Surface area calculations were based on the BET equation, using the software provided. The total pore volume was obtained from the adsorption branches of the isotherms, using the Barrett–Joyner–Halenda (BJH) approach. Each experiment was triplicated.

### 2.10. Differential Scanning Calorimetry (DSC)

The DSC analysis was conducted using a DSC S-650 (Scinco Co. Ltd., Seoul, Korea). Around 2 mg of each sample was accurately weighed, placed in aluminum pans, and sealed. The measurements were carried out at a heating rate of 10 °C/min over a temperature range of 25–80 °C and with a nitrogen-gas purge (flow rate of 40 mL/min). An empty sealed pan was used as the reference sample. The onset temperature of the melting endothermic peak was determined as the intersection point of the tangent of the baseline before the transition and the inflection tangent. The relative crystallinity (RC), which was defined as the relative amount of crystalline orlistat in the samples compared to raw material, was estimated from the area under the orlistat melting peak for the sample compared to that for the raw orlistat crystal as follows [44]:

RC (%) = (C_sample_/C_raw_) × 100, (5)

where RC is the relative percentage of crystalline orlistat in the sample, C_sample_ is the area under the orlistat melting peak for the sample, which was corrected by the measured orlistat amount in the sample based on the drug content analysis result in below method Section 2.13, and C_raw_ is the area under the melting peak for raw orlistat. The detection of onset temperature and integration of melting peaks were performed using Infinity Pro (Scinco Co. Ltd., Seoul, Korea). The DSC measurement for each sample is performed in triplicate.

### 2.11. PXRD

PXRD patterns of samples were obtained using an X-ray diffractometer (Rigaku, D/max-IIIC, Tokyo, Japan). Ni-filtered Cu-Kα line was used as the source of radiation, which was operated at a voltage of 40 kV and a current of 45 mA. Each X-ray diffractogram was recorded from 5° to 50° (2*θ*) at a scanning speed of 5°/min and a step size of 0.05°.

### 2.12. SEM

An SEM (JSM-7000F, JEOL, Japan) was employed for the morphological analysis. The samples were distributed evenly on a metal sample stub using conductive carbon adhesive tape and then coated with a thin layer of gold. The morphology and appearance of samples were examined at an accelerating voltage of 1 or 5 kV.

### 2.13. Analyses of Drug Content (%) and Uniformity

Samples containing about 10 mg of orlistat were weighed accurately and dissolved in ethanol, then diluted to an appropriate concentration for quantification. After filtering through a syringe filter (Whatman, PTFE of 0.45 μm, Clifton, NJ, USA), the sample solution was injected into the HPLC system. Drug content (%) was calculated by dividing the theoretical concentration by the measured concentration and then multiplying by 100. The uniformity of drug content was compared between the amorphisation methods. Based on the USP–NF General Chapter <905> Uniformity of Dosage Units – Solid dosage forms, the acceptance value (AV) was calculated by the following equation [45]:

AV (Acceptance value) = |M − X| + *ks*, (6)

where M is the reference value, X is the mean of individual content (%), *k* is the acceptability (if n = 10, then *k* = 2.4), and *s* is the standard deviation. The target content was 100.0% in this study, and the values of M were as follows: if 98.5% ≤ X ≤ 101.5%, then M = X; if X ≤ 98.5%, then M = 98.5%; and if X ≥ 101.5%, then M = 101.5%. If the AV was less than the L1% = 15%, the content uniformity was acceptable based on the standard criterion of USP. A smaller AV value represented better content homogeneity in the formulation.

### 2.14. In-Vitro Dissolution Study

The dissolution tests of powder samples were carried out using the USP II apparatus with a VK 7000 dissolution testing station and VK 750d heater/circulator (Vankel, Cary, NC, USA). The experiment for each sample was triplicated. Samples containing an equivalent of 60 mg of orlistat were placed in 900 mL of deionised water, containing 1% SLS. The stirring speed was set to 75 rpm, and the temperature was maintained at 37 ± 0.5 °C. At predetermined time points, 2 mL of each sample was drawn and filtered using a 0.45 μm PTFE syringe filter; then, it was appropriately diluted with the dissolution medium. The concentration of orlistat was determined by the HPLC-UV method. The dissolution efficiency (DE) was calculated from the area under the dissolution curve at time t (measured using the trapezoidal rule) and expressed as percentage of the area of the rectangle described by 100% dissolution in the same time. For comparison, raw orlistat and the commercial product, Xenical^TM^ (Roche Pharmaceuticals, Nutley, NJ, USA), were also evaluated.

### 2.15. Statistical Analysis

Statistical analysis was performed by the independent T-test or a one-way analysis of variance (ANOVA) followed by Student–Newman–Keuls (SNK) test, using the SPSS 12.0 software (IBM SPSS, Chicago, IL, USA).

## 3. Results and Discussions

### 3.1. Solubility of Orlistat in SC-CO_2_

**Figure 3:**
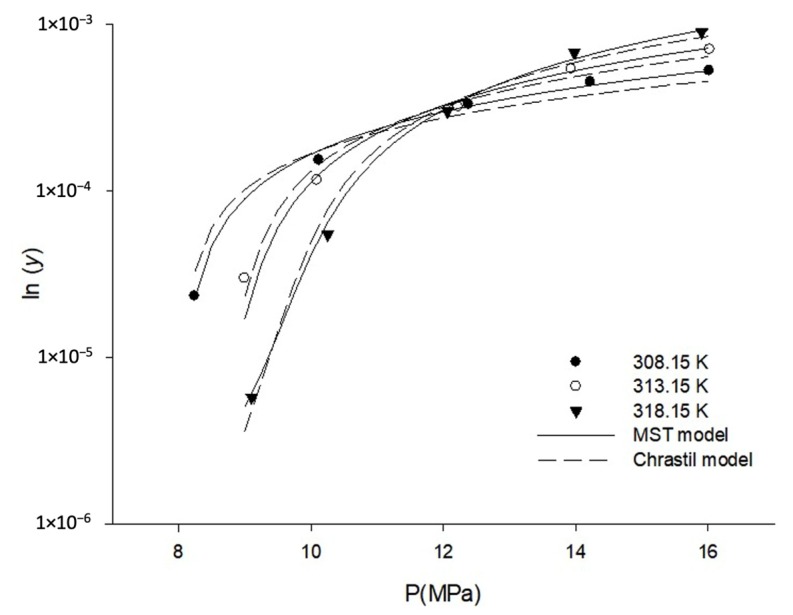
The solubility (mean value, *n* = 3) of orlistat in SC-CO_2_ at various pressure and temperature, measured experimentally and predicted using the two semi-empirical models (Chrastil model and the Mendez-Santiago and Teja (MST) model) to correlate experimental solubility data.

The experimental solubility data, in terms of equilibrium mole fraction, ‘y’, of orlistat and in grams per liter, ‘s’, of orlistat in SC-CO_2_, are summarised in Table 1. The standard deviations (S.D.) for all data points were relatively small (Table 1). In addition, the coefficient of variance, defined as (standard deviation/mean value), for the solid solubility measurements was below 5%. These results indicated the repeatability of our experimental measurements. The solubility of orlistat increased with the increase in pressure, at a constant temperature. When different solubility isotherms were represented in the same plot, the so-called crossover region could be recognised at around 11.8 MPa (Figure 3). This crossover pressure is the result of the competing factors of solute vapor pressure and solvent density. The temperature affects the solubility through these two competing factors. Below this crossover pressure, the solubility of orlistat decreased with increasing temperature because the SC-CO_2_ density effect is dominant. On the other hand, when the pressures are above the crossover region, the effect of solute vapor pressure becomes more dominant, and the SC-CO_2_ density becomes less sensitive to the pressure; hence, the solubility of orlistat increases with temperature [46,47,48,49].

**Figure 4:**
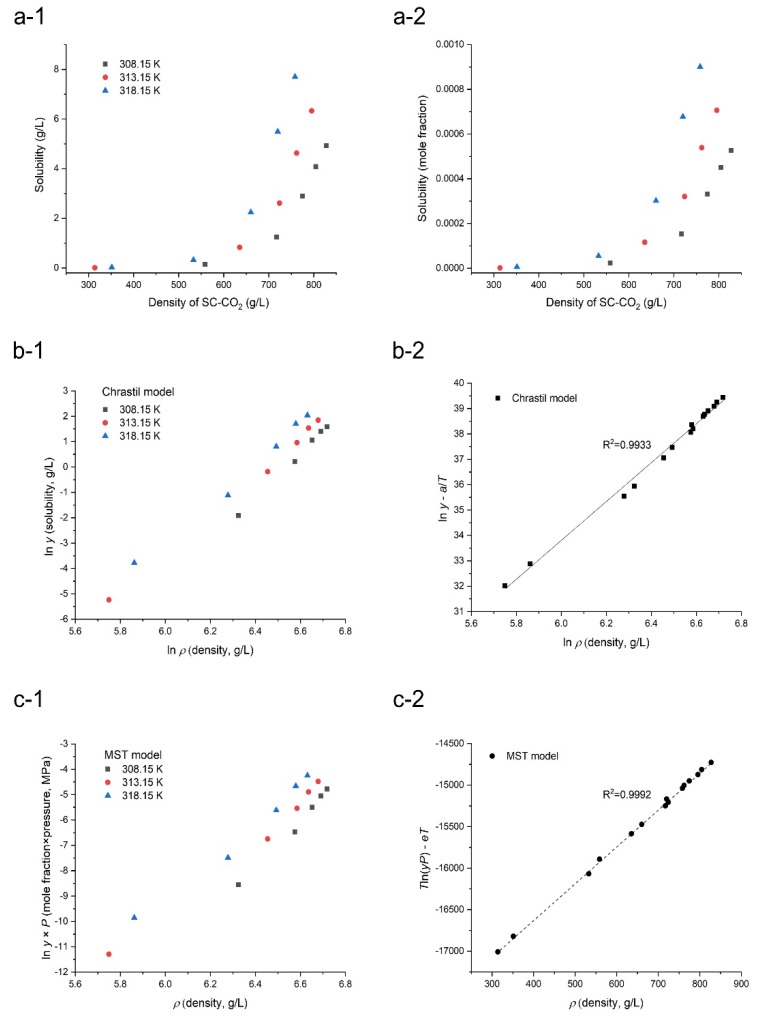
(**a-1**,**a-2**) Dependency of the orlistat solubility (mean value, *n* = 3) on the density of SC-CO_2_ and correlation of orlistat solubility in SC-CO_2_ with two density-based models, (**b-1**,**b-2**) Chrastil (solubility as a unit of g/L) and (**c-1**,**c-2**) MST (solubility as a unit of mole fraction).

The solubility data of orlistat can also be represented in terms of the SC-CO_2_ density, since the effects of both temperature and pressure on the SC-CO_2_ density can be shown together. Table 1 presents the density data of SC-CO_2_, which was obtained from the NIST REFPROP database [41], and the dependences of orlistat solubility with a unit of g/L and mole fraction on the SC-CO_2_ density (g/L) are shown in Figure 4a-1,a-2, respectively. As the density of SC-CO_2_ increased, the solubility of orlistat also increased, thus indicating that the SC-CO_2_ density significantly affected the solubility of orlistat in SC-CO_2_ [50,51]. Interestingly, it was also observed that the solubility of orlistat increased dramatically when the density was higher than 500 g/L. The pronounced temperature effect on the solubility of orlistat is also shown in Figure 4a, which indicated that a higher temperature resulted in higher drug solubility at a constant SC-CO_2_ density. This could be attributed to the fact that a solute has a higher vapor pressure at a higher temperature.

The solubility data can be conveniently correlated with the density of SC-CO_2_. Among the several empirical density-based correlations proposed previously, two models presented by Chrastil and MST, respectively were applied in this study. As presented in Equations (1) and (2), the two empirical models showed a dependence of ln*y* on 1/*T* due to the close relation between the solubility and the solute vapor pressure, besides a dominant dependence of the drug solubility on the solvent density. The linear correlations were observed between ln*y* versus ln *ρ* and between *T*ln(*yP*) versus *ρ*, based on the Chrastil and MST models, respectively (Figure 4b-1,c-1). The increase in solubility with increase of SC-CO_2_ density and temperature was confirmed once again. Furthermore, the correlations obtained using the Chrastil and MST models were also plotted as ln*y* - *a/T* versus ln *ρ*, based on Equation (1) and *T*ln(*yP*) - *eT* vs. *ρ*, based on Equation (2), and these are presented in Figure 4b-2,c-2. This treatment is advantageous since it combines the temperature effect into the solubility term and subsequently results in an obvious linear dependence of solubility on solvent density, and all the solubility data at different temperatures and pressures coincide to form a single straight line. As proposed by Méndez-Santiago and Teja, the self-check of data could be readily done through the examination of the experimental point deviation from the linear regression line. As shown in Figure 4c, most of the data points condensed to a specific straight line rather well for both Chrastil and MST models, and the solubility was observed to increase monotonically and almost linearly with SC-CO_2_ density. Furthermore, the predicted orlistat solubility data, determined by the two empirical models, are presented in Figure 3, which shows that the predicted solubility was similar to the experimental solubility. However, some data points were sparsely scattered around the correlation line, which is indicative of the poor solubility prediction of the used model. This scatter of data, which implied inconsistency between predicted and experimentally obtained data, can be presented as AARD [52]. The obtained correlation parameters and the AARD of the two empirical models to correlate experimental solubility data are demonstrated in Table 4. The AARD values of orlistat were 16.67 and 6.37 for Chrastil and MST models, respectively. In addition, the R^2^ value of the linear regression line was closer to 1 in the MST model than in the Chrastil model (Figure 4b-2,c-2). These results confirmed that the MST model was a better fit than the Chrastil model and could predict the solubility of orlistat better than the Chrastil model. It has been reported that the MST model produces much more reliable, correlated results for solubility data at different pressures, since it also includes an individual pressure term.

To better understand the loading behavior of orlistat onto mesoporous silica in SC-CO_2_ and establish proper operating conditions for the MA-SCF process, it is vital to determine the solubility of orlistat under various pressure and temperature equilibrium conditions and to correlate the solubility data with a well-established model. Thus, the obtained correlation between SC-CO_2_ density and orlistat solubility, with pressure and temperature changes, will be discussed in more detail for a better understanding of the changes in loading behavior due to process variables, such as temperature and pressure.

### 3.2. Melting Point Depression of Orlistat

To observe the change in melting temperature with increasing pressure and establish the range of MA-SCF operating conditions that could utilise the amorphisation using the melting phenomenon, a phase behavior study was conducted to determine the melting point depression of orlistat by SC-CO_2_. Experimentally measured values of the melting temperature of orlistat in the presence of SC-CO_2_ are shown in Table 2. The relative small S.D. and the coefficient of variance of less than 3% for measured all melting pressure data (Table 2) showed the repeatability of our experimental measurements. It was observed that the melting point (T_m_) of orlistat decreased significantly with increasing pressure range from 0.1 to 5 MPa. It has been reported that gases with high solubility can decrease the melting points considerably, whereas gases with small solubility lead to an increase in the melting points [42,53]. Considering this, it can be assumed that the melting point of orlistat decreased in SC-CO_2_. The results of solubility of orlistat in SC-CO_2_ and melting point depression presented in this work can provide essential information to determine the operating conditions for the MA-SCF process. Hence, these results will be discussed in detail in the later sections to explain the change in the loading characteristics of molten orlistat with pressure and temperature changes.

### 3.3. Effects of Pressure on the Orlistat Loading onto Mesoporous Silica

**Figure 5:**
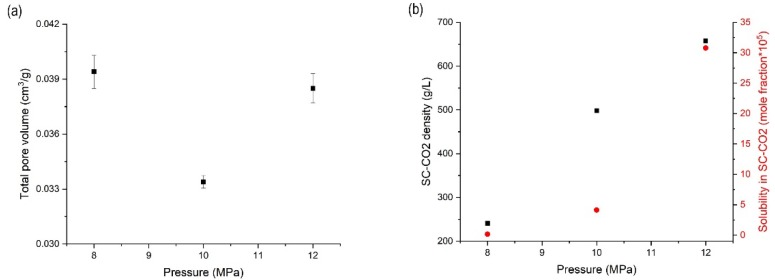
(**a**) Change in total pore volume with increasing pressure at 318.15 K and (**b**) the correlation of pressure to SC-CO_2_ density and solubility of orlistat in SC-CO_2_.

The RC (%), total pore volume, and dissolution (DE_10_, %) results of orlistat-loaded Neusilin^®^ UFL2 samples, prepared by MA-SCF using the process conditions described in this study, are summarized in Table 3. Samples were prepared at 8, 10, and 12 MPa, with a constant temperature of 318.15 K. Various characterisation methods were performed to understand the effect of the change in pressure on the results. When the pressure increased from 8 to 10 MPa, the total pore volume of the sample decreased. Subsequently, when the pressure was increased up to 12 MPa, the total pore volume increased inversely (Figure 5a). This complicated trend could be explained by the density of SC-CO_2_ and the solubility of orlistat in SC-CO_2_. The density of SC-CO_2_ increased with increasing pressure (Figure 5b) and this high density SC-CO_2_, which could freely penetrate into molten orlistat, facilitated the introduction of orlistat into the pores of Neusilin^®^UFL2. In other words, the high concentration of SC-CO_2_ in the molten liquid phase of orlistat led to a considerable reduction in viscosity and interfacial tension, which in turn allowed the penetration of orlistat into the pores of Neusilin^®^UFL2. However, when the solubility of orlistat increased excessively by increasing the density of SC-CO_2_, the orlistat dissolved in SC-CO_2_ might have been released from the pores of Neusilin^®^UFL2 during the MA-SCF process. As shown in Figure 5b, the solubility of orlistat increased dramatically when the pressure was increased beyond 10 MPa with a density higher than 500 g/L. Thus, it can be estimated that the entrapment of orlistat in pores decreased after 10 MPa; hence, the pore volume increased.

**Figure 6:**
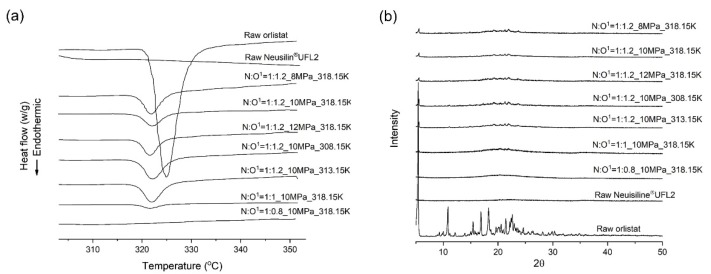
(**a**) DSC thermograms and (**b**) powder X-ray diffraction (PXRD) patterns for raw materials and orlistat-loaded Neusilin^®^UFL2 samples prepared by MA-SCF at various pressure and temperature.^1^N:O is the mass ratio of Neusilin^®^UFL2:Orlistat.

DSC thermograms and PXRD patterns for raw materials and orlistat-loaded Neusilin^®^UFL2 samples, prepared by MA-SCF at various pressure and temperature, are shown in Figure 6. Raw orlistat crystals were characterised by a single sharp melting endothermic peak, with a peak temperature of 324.75 K (ΔH = 76.37 J/g) and onset temperature of 321.05 K, during the DSC analysis. The characteristic diffraction peaks due to the orlistat crystalline structure were observed at 2θ of 5.4°, 10.8°, 15.5°, 16°, 18.1°, 21.4°, and 22.6° in the PXRD pattern, which corresponded to the previously reported melting temperature and PXRD pattern for pure crystalline orlistat [54].

**Figure 7:**
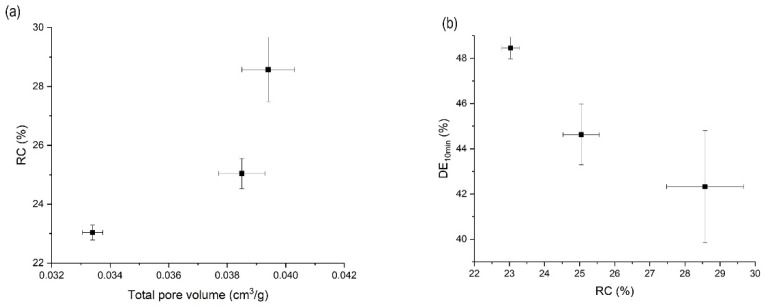
(**a**) Correlation between total pore volume and relative crystallinity (RC) and (**b**) correlation between RC and DE_10_ for orlistat-loaded Neusilin^®^UFL2 samples prepared by MA-SCF at various pressure with a constant temperature of 318.15 K.

The DSC thermograms showed that all samples prepared by the MA-SCF method had smaller melting enthalpy compared to that of raw orlistat, and the halo of the background appeared clearly with very weak crystalline peaks in the PXRD patterns. Thus, it was estimated that a small amount of the crystalline form coexists with a large amount of the amorphous form in the samples. Several researchers have reported that a drug exists in the amorphous form when it is distributed in the pores of a mesoporous silica [55,56,57]. Interestingly, as the total pore volume increased, the RC obtained from DSC also increased (Figure 7a). These results indicate that the crystalline portion in the prepared formulation is mainly composed of drugs existing outside the pores of Neusilin^®^UFL2 [58]. The overall dissolution profiles of the prepared samples are shown in Figure S2 (Supplementary Material), and it was observed that dissolution rate decreased with increasing crystallinity (Table 3 and Figure 7b). This trend of change in the dissolution rate due to crystallinity is well known. The disruption of the crystal lattice by a solvent requires input of energy during the dissolution process. However, amorphous systems do not require the breakage of the crystal lattice, and they have a solubility advantage compared to the solid crystalline forms. Hence, lower crystallinity results in a higher dissolution rate [59].

From the above results, it was shown that the orlistat-loaded Neusilin^®^ UFL2 sample prepared at 10 MPa among samples prepared at various pressures with a fixed temperature of 318.15 K had the most desirable pharmaceutical properties with low crystallinity and a high dissolution rate.

### 3.4. Effects of Temperature on the Orlistat Loading onto Mesoporous Silica

**Figure 8:**
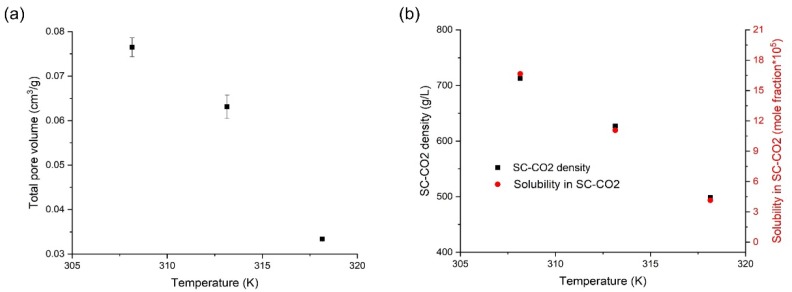
(**a**) Change in total pore volume with increasing temperature at 10 MPa and (**b**) the correlation of temperature with SC-CO_2_ density and solubility of orlistat in SC-CO_2_.

Orlistat-loaded Neusilin^®^UFL2 samples were prepared under different temperatures at 10 MPa, ranging from 308.15 to 318.15 K, to observe the effect of change in temperature on the resultant formulation. As shown in Figure 8a,b, the pore volume was the largest at 308.15 K; then, it decreased with increasing temperature up to 318.15 K, presumably because the solubility of orlistat in SC-CO_2_ decreased, which in turn reduced the amount of drug released from the pore; therefore, the incorporation of orlistat inside the pores increased. In addition, as revealed in the above results on the effect of pressure, it was confirmed again that the total pore volume decreased when the SC-CO_2_ density decreased close to 500 g/L. However, typically, a decrease in density of SC-CO_2_ led to an increase in viscosity of the molten drug [31,38]. Thus, it is presumed that the increase in temperature from 308.15 to 318.15 K at 10 MPa itself results in decrease in viscosity of molten orlistat, and this effect counteracts the increase in the viscosity of the molten orlistat by SC-CO_2_ density reduction. From these results, it is proposed that the synergic effect of the above two positive factors, decrease in orlistat solubility in SC-CO_2_, and decrease in viscosity could play a major role in increasing the orlistat loading efficiency by increase in temperature.

**Figure 9:**
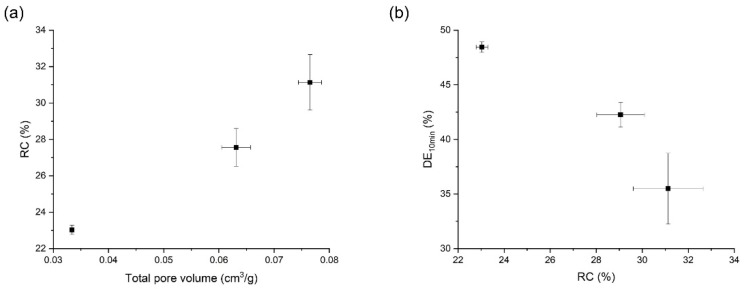
(**a**) Correlation between total pore volume and RC and (**b**) correlation between RC and DE_10_ for orlistat-loaded Neusilin^®^UFL2 samples prepared by MA-SCF at various temperature, with a constant pressure of 10 MPa.

**Figure 10:**
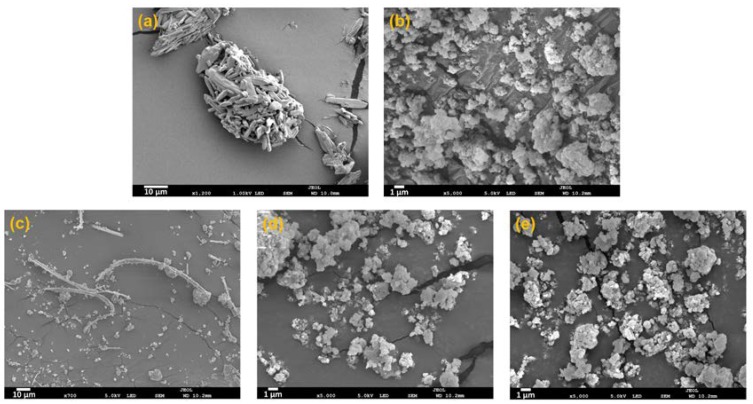
SEM images: (**a**) raw orlistat, (**b**) raw Neusilin^®^ UFL2, Neusilin^®^UFL2: orlistat = 1:1.2 (*w/w*) samples prepared by MA-SCF at (**c**) 308.15 K and 10 MPa and (**d**) 318.15 K and 10 MPa, and (**e**) Neusilin^®^UFL2: orlistat = 1:0.8 (*w/w*) sample prepared by MA-SCF at 318.15 K and 10 MPa.

All samples prepared by the MA-SCF method at various temperatures with a fixed pressure of 10 MPa had smaller melting enthalpies as compared to raw orlistat in the DSC thermograms, and the halo of the background appeared clearly with very weak crystalline peaks in the PXRD patterns (Figure 6). This indicated the coexistence of a small amount of crystalline form with a large amount of amorphous form in the prepared samples. As expected from the results on the effect of pressure in Section 3.3, the RC increased with increasing pore volume (Figure 9a) and the dissolution rate decreased with increasing crystallinity (Figure 9b). Interestingly, the sample prepared at 308.15 K, with the largest total pore volume, showed crystals with long string-like shapes separated from the Neusilin^®^UFL2, as observed in SEM images (Figure 10). The shape of these particles may also show relatively high crystallinity.

There results indicate that the orlistat-loaded Neusilin^®^ UFL2 sample prepared at 318.15 K among samples prepared at various temperatures with a fixed pressure of 10 MPa had the most desirable pharmaceutical properties with high drug loading, low crystallinity, and high dissolution rate.

### 3.5. Effects of Mesoporous Silica:Drug Mass Ratio on the Orlistat Loading Behavior onto Mesoporous Silica

**Figure 11:**
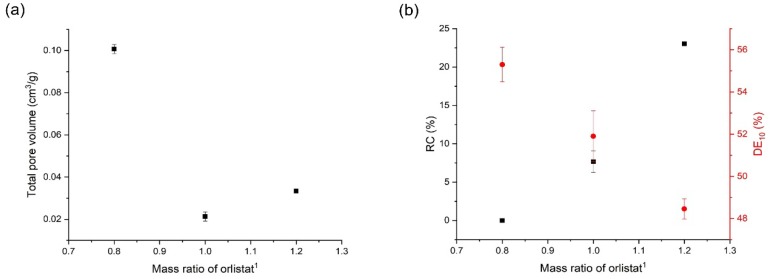
(**a**) Change in total pore volume with change in mass ratio of orlistat and (**b**) the correlation of orlistat mass ratio with relative crystallinity (RC) and DE_10_. ^1^ The relative mass ratio of orlistat to Neusilin^®^UFL2 obtained by dividing the mass of orlistat by the mass of Neusilin^®^UFL2.

The results from the evaluation tests of orlistat-loaded Neusilin^®^UFL2 with different mass ratios, prepared by the MA-SCF method, are presented in Table 3. The mass ratios of Neusilin^®^UFL2:orlistat were 1:1.2, 1:1, and 1:0.8 (*w/w*). Figure 11a shows that the lowest total pore volume was obtained near a 1:1 mass ratio. This result indicates that at 1.2 mass ratio of orlistat, the drug was in excess of the drug loading capacity of Neusilin^®^UFL2. In contrast, the highest total pore volume at a 0.8 mass ratio of orlistat probably indicated a void space left after loading of almost all the orlistat onto Neusilin^®^UFL2. The results from the DSC and PXRD analyses suggest that as the mass ratio of orlistat decreased from 1.2 to 0.8, the crystallinity continued to decrease, eventually reaching the amorphous form at 0.8 (Figure 6 and Figure 11b). This result confirms the above-mentioned assumption that the orlistat filled in the pores of Neusilin^®^UFL2 are amorphous, while orlistat distributed outside the pore exists mainly in crystalline form (Figure 11b). From these results, it can be predicted that formulations with a minimum total pore volume of orlistat-loaded Neusilin^®^UFL2 by MA-SCF, which means that the orlistat is maximally filled in the pore, can be produced at a relative mass ratio of orlistat between 1 and 0.8 compared to Neusilin^®^UFL2. This experimentally obtained an optimal loaded orlistat mass ratio compared to Neusilin^®^UFL2, which is lower than 1.2 and is close to the theoretical optimal orlistat loading mass ratio estimated based on the experimentally measured pore volume of Neusilin^®^UFL2 and the true density of orlistat. The reason for this deviation may be due to the expanded volume of molten orlistat in which the SC-CO_2_ penetrated, compared to the crystalline state [60].

The dissolution rate increased with the decrease in crystallinity due to the decrease in the mass ratio of orlistat (Figure 11b). Therefore, obtaining the optimal ratio of the drug to mesoporous silica is crucial in the MA-SCF method to achieve efficient dissolution of the drug. From these results, it was shown that the most desirable mass ratio of Neusilin^®^UFL2:orlistat for the amorphisation is 1:0.8 among samples prepared at various mass ratio in this study.

### 3.6. Comparison of Amorphisation Method

To determine the effect of the amorphisation method, orlistat-loaded Neusilin^®^UFL2 was also prepared by the SE and HM methods at a mass ratio of Neusilin^®^UFL2:orlistat = 1:0.8 (*w/w*), and this orlistat-loaded mesoporous silica was compared to the formulation with the best outcome prepared by the MA-SCF method at 318 K and 10 MPa. Results from the uniformity analysis (Table 5) showed that the AV value decreased in the following order: HM > SE > MA-SCF (ANOVA, *p* < 0.05, ranked by the SNK test), thus indicating that the uniformity increased in the reverse order.

**Figure 12:**
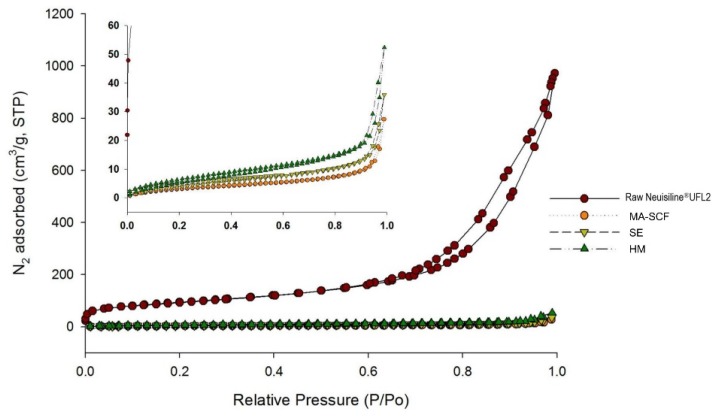
Nitrogen adsorption–desorption isotherms of raw Neusilin^®^UFL2 and orlistat-loaded Neusilin^®^UFL2 prepared by various amorphisation methods.

**Figure 13:**
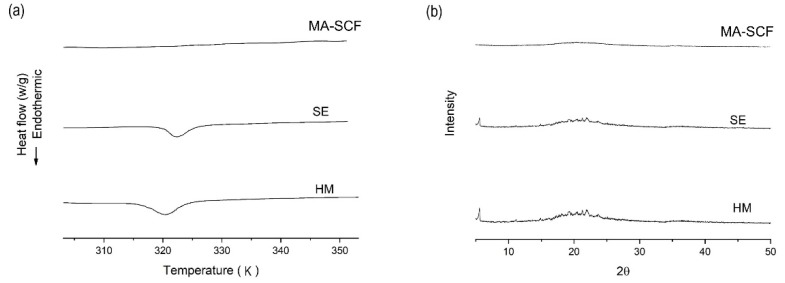
(**a**) DSC thermograms (**b**) and PXRD patterns for raw materials and orlistat-loaded Neusilin^®^UFL2 samples prepared by various amorphisation methods.

Further, nitrogen adsorption decreased in the following order: raw Neusilin^®^UFL2 > HM > SE > MA-SCF (Figure 12). It was observed that nitrogen adsorption of raw Neusilin^®^UFL2 could be decreased after the loading of orlistat onto Neusilin^®^UFL2 by various methods. This is probably because the large surface area onto which nitrogen could be adsorbed was reduced by the filling orlistat into the pores of Neusilin^®^UFL2. The higher nitrogen adsorption indicated a larger surface area, which implied that the concentration of orlistat filled inside pore was low. Thus, the higher loading efficiency of orlistat onto Neusilin^®^UFL2 would result in the reverse order of nitrogen adsorption of prepared sample. It can be assumed that for the HM method, the introduction of orlistat into the pores could also be inhibited due to the high viscosity of molten orlistat [61]. For the sample prepared by the SE method, the introduction of orlistat deep inside the pores of Neusilin^®^UFL2 would be reduced by the low diffusivity, high velocity, and disturbance of wettability, as well as by the surface tension of the organic solvent [62]. On the contrary, the high diffusivity and low viscosity of SC-CO_2_ might have contributed to the higher loading efficiency of orlistat onto Neusilin^®^UFL2. The crystallinity decreased in the following order: HM > SE > MA-SCF (Table 5 and Figure 13). Only the sample prepared by the MA-SCF method was in a pure amorphous state. It was confirmed once again that the lower the drug-loading efficiency inside the pore of mesoporous silica, the higher the crystallinity of the prepared formulation. This was apparently because the orlistat that solidified outside the pore precipitated to mainly a crystalline form, as mentioned above [58,63].

The percentages of the drug dissolved in 10 and 30 min were used to compare the dissolution rate for raw orlistat, the commercial product, and the powders prepared by the various amorphisation methods. As shown in Table 5, all formulations prepared by various amorphisation methods showed faster dissolution than raw orlistat and the commercial product. The faster dissolution rate of orlistat in the formulations prepared by the three amorphisation methods could be attributed to the increased surface area of orlistat after loading onto Neusilin^®^UFL2 and change from the crystalline to amorphous form. In particular, the formulation prepared by the MA-SCF method had the highest dissolution rates among the prepared powders using various amorphisation methods, and it showed a faster dissolution rate than the raw orlistat and commercial product, with approximately 8.6-fold and 5.9-fold increases in DE_60_, respectively. This result is probably because the samples prepared by the SE and HM methods exist as mixtures of crystalline and amorphous forms, whereas the formulation prepared by the MA-SCF method is in a pure amorphous form because of the efficient loading of orlistat into the pores of Neusilin^®^UFL2.

## 4. Conclusions

In this study, orlistat-loaded mesoporous silica was successfully prepared using the MA-SCF method to obtain a formulation with an improved dissolution rate of orlistat. In addition, the effects of pressure and temperature during the MA-SCF process on the properties of the prepared orlistat formulation were investigated. To explain the effect of these process parameters, the correlations of SC-CO_2_ density and drug solubility in SC-CO_2_ with pressure and temperature changes were studied using semi-empirical models. Thus, the correlations obtained could explain the differences in the loading behavior of orlistat onto the porous solid carrier (Neusilin^®^UFL2) due to process variables such as temperature and pressure. The results showed that the total pore volume of orlistat-loaded mesoporous silica decreased with the increasing density of SC-CO_2_. From this tendency, it is suggested that the high density of SC-CO_2_, which could penetrate into molten orlistat and decrease the viscosity, facilitated the loading of orlistat into the pores of Neusilin^®^UFL2. However, when the density of SC-CO_2_ increased to more than 500 g/L, the total pore volume increased, which might be due to an increased solubility of orlistat in SC-CO_2_. This suggested that the amount of orlistat dissolved in SC-CO_2_ might be released from the pores of Neusilin^®^UFL2 during the MA-SCF process. As the total pore volume of the prepared sample increased, the crystallinity also increased, which indicated that the orlistat that filled the pores of Neusilin^®^UFL2 could exist in an amorphous state; in contrast, the crystalline portion of the prepared formulation might be mainly composed of drugs existing outside pores. The dissolution rate decreased with increasing crystallinity. In addition, it was shown that the mass ratio of the drug to the mesoporous silica is an important factor in the MA-SCF method to achieve an efficient improvement in the dissolution of the drug via amorphisation. Thus, in order to prepare the amorphous drug-loaded solid carrier formulation with the desired properties using the MA-SCF process, it is important to identify a specific range of process conditions in which the density of SC-CO_2_ is relatively high and the solubility of orlistat in SC-CO_2_ is relatively low, which can result in the lowest total pore volume (the highest drug-loading effiency), crystallinity, and the highest dissolution rate. Furthermore, a comparison of the various amorphisation methods showed that the MA-SCF method was the most suitable method for producing uniform amorphous dispersions in which the drug was loaded onto the mesoporous carrier and had improved dissolution.

## Figures and Tables

**Table 1:** Solubilities of orlistat in SC-CO_2_ (*n* = 3).

Temperature (K)	Pressure (MPa)	CO_2_ Density (g/L) [41]	Solubility of Orlistat ± S.D. *y* (Mole Fraction) × 10^5^	s (Mean Value, g/L = kg/m^3^)
308.15	8.25	558.62	2.3 ± 0.1	0.15
	10.12	717.2	15.3 ± 0.2	1.24
	12.38	774.62	33.1 ± 1.1	2.89
	14.23	804.64	45.0 ± 1.4	4.08
	16.02	827.4	52.7 ± 1.2	4.92
313.15	8.28	314.02	0.2 ± 0.0	0.01
	10.10	635.51	11.6 ± 0.2	0.83
	12.23	724.12	32.0 ± 0.9	2.61
	13.94	762.06	53.9 ± 1.5	4.63
	16.03	795.28	70.6 ± 2.6	6.33
318.15	9.10	351.49	0.6 ± 0.0	0.02
	10.25	532.91	5.5 ± 0.1	0.33
	12.06	660.27	30.1 ± 0.6	2.24
	13.98	719.99	67.7 ± 2.7	5.50
	15.90	758.35	90.1 ± 3.5	7.70

**Table 2:** Melting point depression of orlistat in a binary system with CO_2_ observed by solid–liquid (S-L) phase behavior experiment (*n* = 3).

Binary System (CO_2_ + Orliastat)		
Pressure (MPa) ^1^ ± S.D.	T_m_p_ (K) ^2^	ΔT_m_ (K) ^4^
Ambient	321.05 ^3^	-
2.3 ± 0.1	313.15	−8
3.2 ± 0.1	308.15	−13
3.7 ± 0.1	303.15	−18
4.0 ± 0.1	298.15	−23
4.3 ± 0.1	293.15	−28
4.8 ± 0.1	288.15	−33

^1,2^ The melting pressure and temperature of orlistat were determined by visual observation during the S-L phase behavior experiment. ^3^ The onset temperature obtained from the melting endothermic peak in differential scanning calorimetry (DSC) thermogram of raw orlistat at ambient pressure. ^4^ The melting point depression was calculated using Equation (4).

**Table 3:** Experimental conditions of the melt-amorphisation by supercritical fluid (MA-SCF) process.

Factors			Results (*n* = 3, mean ± S.D.)			
Mass Ratio (N:O) ^1^	T (K) ^2^	P (MPa) ^3^	Total Pore Volume (cm^3^/g)	Drug Content (%)	RC (%) ^4^	DE_10_ (%)^5^
1:1.2	318.15	8	0.0394 ± 0.0009	97.5 ± 1.2	29.6 ± 1.1	42.3 ± 2.5
1:1.2	318.15	10	0.0334 ± 0.0004	97.7 ± 1.1	23.0 ± 0.3	48.5 ± 0.5
1:1.2	318.15	12	0.0385 ± 0.0008	93.4 ± 2.1	25.0 ± 0.5	44.6 ± 1.3
1:1.2	308.15	10	0.0765 ± 0.0021	94.4 ± 0.9	31.1 ± 1.5	35.5 ± 3.3
1:1.2	313.15	10	0.0631 ± 0.0026	97.2 ± 2.5	29.1 ± 1.0	42.3 ± 1.1
1:1	318.15	10	0.0213 ± 0.0021	98.6 ± 1.3	7.7 ± 1.4	51.9 ± 1.2
1:0.8	318.15	10	0.1007 ± 0.0022	99.8 ± 1.0	0.00 ± 0.0	55.3 ± 0.8

^1^ Mass ratio of Neusilin^®^UFL2: orlistat. ^2^ The process temperature. ^3^ The process pressure. ^4^ The relative crystallinity compared to raw orlistat obtained from DSC analysis. ^5^ The dissolution efficiency after 10 min of the dissolution test.

**Table 4:** Correlation parameters and average absolute relative deviations (AARDs) of the two semi-empirical models (Chrastil model and MST model) to correlate experimental solubility data.

Model	Parameters	AARD (%)
Chrastil	*k* = 7.727, *a* = −11,663.6, *b* = −12.61	16.67
Méndez -Santiago and Teja (MST)	*c* = −18,341.6, *d* = 4.369, *e* = 43.018	6.37

**Table 5:** Evaluated pharmaceutical characteristics of orlistat-loaded Neusilin^®^UFL2 prepared by various methods.

Method	RC (%) ^1^	% Drug Dissolved ^2^		DE_60_ (%) ^3^	Content (%) ^4^	AV (%) ^5^
		10 min	30 min			
Raw Orlistat	100	7.0 ± 1.1	10.2 ± 0.9	9.7 ± 1.1	-	-
Commercial Product	-	7.9 ± 2.1	15.3 ± 1.4	14.1 ± 1.3	-	-
MA-SCF	0	77.0 ± 0.7	89.8 ± 0.8	83.0 ± 1.1	99.8 ± 1.0	2.4
SE	17.4	65.5 ± 1.0	75.8 ± 1.2	70.8 ± 1.9	100.5 ± 3.6	8.7
HM	24.8	57.2 ± 1.9	66.2 ± 1.8	61.9 ± 3.3	98.5 ± 7.1	17.0

^1^ The relative crystallinity (RC) compared to raw orlistat obtained from DSC analysis. ^2^ The percentage of dissolved orlistat after 10 and 30 min of the dissolution test. ^3^ The dissolution efficiency after 60 min of the dissolution test. ^4^ Drug content (%) was calculated by dividing the theoretical concentration by the measured concentration and then multiplying by 100. ^5^ Acceptance value (AV) calculated by Equation (6), in which the smaller AV value represented the better homogeneity of orlistat content in the formulation.

## Supplementary Materials

The following are available online at https://www.mdpi.com/1999-4923/12/4/377/s1, Figure S1: Comparison of the solubility data for fenofibrate in supercritical carbon dioxide at 318.2 K reported in the literature and this work, Figure S2: Dissolution profiles of orlistat-loaded Neusilin^®^UFL2: (a) effect of pressure; (b) effect of temperature; and (c) effect of orlistat mass ratio for MA-SCF process; and (d) effect of preparation method. 1N:O is the mass ratio of Neusilin^®^UFL2:Orlistat.
