# Supplementary material for: Melt Amorphisation of Orlistat with Mesoporous Silica Using a Supercritical Carbon Dioxide: Effects of Pressure, Temperature, and Drug Loading Ratio and Comparison with Other Conventional Amorphisation Methods
Source: Pharmaceutics. 2020 Apr 20;12(4):377. doi: 10.3390/pharmaceutics12040377 (PMC7238234; doi:10.3390/pharmaceutics12040377)
Supplement: Supplementary file 1 [file pharmaceutics-12-00377-s001.pdf]

pharmaceutics

Supplementary Materials: Melt Amorphisation of Orlistat with Mesoporous Silica using a Supercritical Carbon Dioxide: Effects of Pressure, Temperature, and Drug Loading Ratio and Comparison with other Conventional Amorphisation Methods

Heejun Park, Kwang-Ho Cha, Seung Hyeon Hong, Sharif Md Abuzar, Eun-Sol Ha, Jeong-Soo Kim, Min-Soo Kim and Sung-Joo Hwang

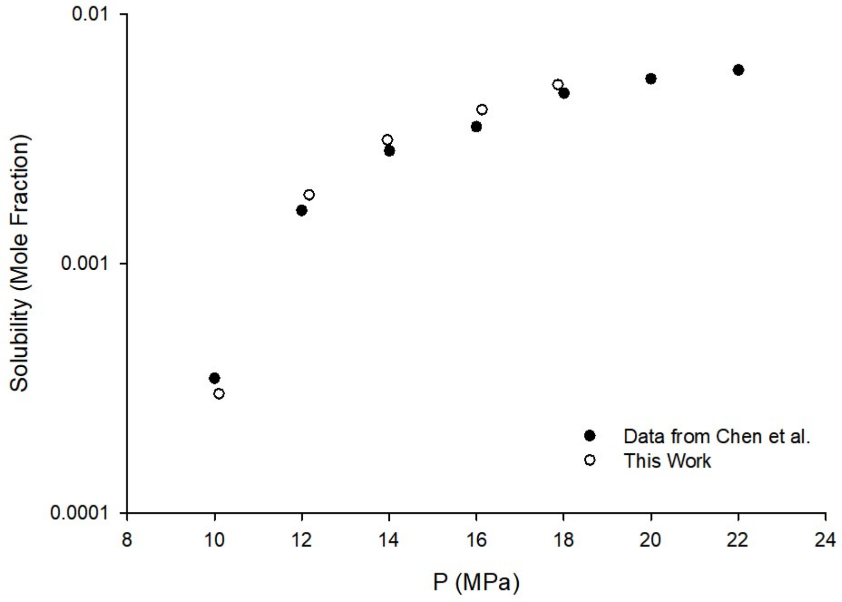

Figure S1. Comparison of the solubility data for fenofibrate in SC-CO_2 at 318.15 K reported in the literature [40] and this work.

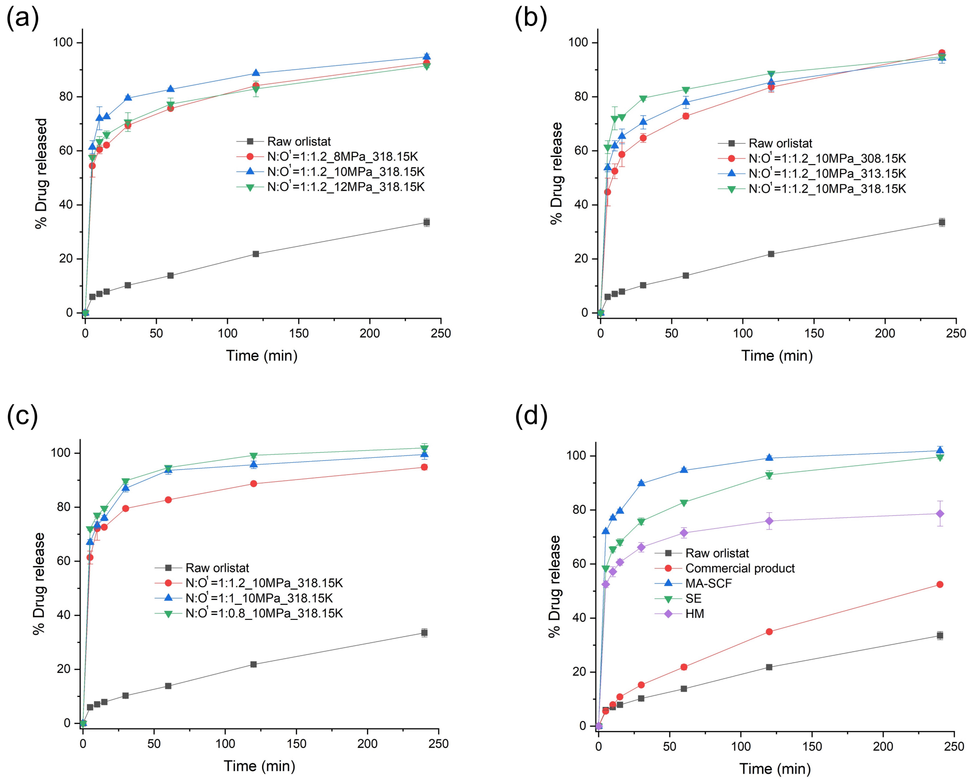

Figure S2. Dissolution profiles of orlistat-loaded Neusilin®UFL2: (**a**) effect of pressure; (**b**) effect of temperature; and (**c**) effect of orlistat mass ratio for MA-SCF process; and (**d**) effect of preparation method. [1]N:O is the mass ratio of Neusilin®UFL2:Orlistat.
